# Embodied intersubjective engagement in mother–infant tactile communication: a cross-cultural study of Japanese and Scottish mother–infant behaviors during infant pick-up

**DOI:** 10.3389/fpsyg.2015.00066

**Published:** 2015-02-27

**Authors:** Koichi Negayama, Jonathan T. Delafield-Butt, Keiko Momose, Konomi Ishijima, Noriko Kawahara, Erin J. Lux, Andrew Murphy, Konstantinos Kaliarntas

**Affiliations:** ^1^Faculty of Human Sciences, Waseda UniversityTokorozawa, Japan; ^2^Faculty of Humanities and Social Sciences, University of StrathclydeGlasgow, UK; ^3^Faculty of Home Economics, Kyoritsu Women’s UniversityTokyo, Japan; ^4^Department of Biomedical Engineering, University of StrathclydeGlasgow, UK; ^5^School of Life, Sport and Social Sciences, Edinburgh Napier UniversityEdinburgh, UK

**Keywords:** embodied intersubjectivity, cultural learning, development, Japan and Scotland, mother–infant relations, motor control, anticipation, peri-personal space

## Abstract

This study examines the early development of cultural differences in a simple, embodied, and intersubjective engagement between mothers putting down, picking up, and carrying their infants between Japan and Scotland. Eleven Japanese and ten Scottish mothers with their 6- and then 9-month-old infants participated. Video and motion analyses were employed to measure motor patterns of the mothers’ approach to their infants, as well as their infants’ collaborative responses during put-down, pick-up, and carry phases. Japanese and Scottish mothers approached their infants with different styles and their infants responded differently to the short duration of separation during the trial. A greeting-like behavior of the arms and hands was prevalent in the Scottish mothers’ approach, but not in the Japanese mothers’ approach. Japanese mothers typically kneeled before making the final reach to pick-up their children, giving a closer, apparently gentler final approach of the torso than Scottish mothers, who bent at the waist with larger movements of the torso. Measures of the gap closure between the mothers’ hands to their infants’ heads revealed variably longer duration and distance gap closures with greater velocity by the Scottish mothers than by the Japanese mothers. Further, the sequence of Japanese mothers’ body actions on approach, contact, pick-up, and hold was more coordinated at 6 months than at 9 months. Scottish mothers were generally more variable on approach. Measures of infant participation and expressivity indicate more active participation in the negotiation during the separation and pick-up phases by Scottish infants. Thus, this paper demonstrates a culturally different onset of development of joint attention in pick-up. These differences reflect cultures of everyday interaction.

## INTRODUCTION

Human culture is marked by social expectation and patterns of engagement. Differences in culture are constituted by implicit differences in patterns and style of social expectation and engagement, as well as explicit differences in adornment, and language, and differences in value. For example, greeting styles are composed not only of the gestural codes of, e.g., bowing in Japan or hand-shaking in Scotland, but they differ markedly in their course of social expectation and engagement constituted by psychological values that places one’s actions correctly within an acceptable cultural context. These expectations produce its cultural narrative (Bruner, 1990). Such implicit knowledge of expectation and patterns of affect, arousal, and interest co-regulation is an important contributing element of learned cultural knowledge (Merker, 2009; Frank and Trevarthen, 2012).

Patterns of engagement are learned in embodied social interaction in infancy from birth (Delafield-Butt and Trevarthen, 2013; Trevarthen and Delafield-Butt, 2013; Kugiumutzakis and Trevarthen, 2015). At this early age, infants are able to guide their movements purposefully to achieve desired sensory effects (van der Meer et al., 1995), including social responses from caregivers (Nagy and Molnar, 2004). Such early self-generated action made with anticipation of its sensory contingencies is a fundamental marker of intentionality, and it is expressed in early life before conceptual and reflective development has become established to give a primary form of intentionality (Delafield-Butt and Gangopadhyay, 2013). Primary intentional actions generate sensory consequences that give knowledge of the world, and while the origins of intention has been controversial in psychology (Zeedyk, 1996), it is clear that infants actively contribute to social engagements and learn from these to anticipate their outcomes, within primary experience (Trevarthen and Reddy, 2007; Gallagher, 2008; Trevarthen, 2009; Panksepp, 2011).

Self-generated action repeated regularly over cycles of activity – in what Baldwin (1895) called the ‘circular reaction’ – forms the basis of knowledge and understanding, generating reliable patterns, or ‘schemas,’ of sensorimotor knowledge (Piaget, 1953, 1954; Trevarthen and Delafield-Butt, 2015). Shared between persons, regularly patterned acts of common purpose form the foundation of cultural understanding, they co-create meaning (Delafield-Butt and Trevarthen, 2013). These regular action patterns and their exchange of the motives and feelings that guide them form the basis of an intersubjective, socially generated and embodied knowledge of a culture (Donaldson, 1978; Halliday, 1978; Bruner, 1996; Rogoff, 2003; Legerstee, 2005; Gratier and Trevarthen, 2008; Reddy, 2008; Frank and Trevarthen, 2012).

Even everyday embodied interactions during practical tasks enable communication of expectation with their affects and intentions made manifest within simple acts, such as picking up the infant for feeding. Self-other intentionality within these acts can be read by direct neural resonance of their motor patterns (Gallese, 2003; Ammaniti and Gallese, 2014), giving implicit meaning within a ‘direct’ and intrinsically ‘smart’ social perception (Gallagher, 2008). Seminal psychologist Daniel Stern recognized these bodily projects are patterned with narrative form structured by their intended outcome, which enables learning the consequences of expression in social projects (Stern, 1985). Intimate engagements attuned to each other’s affects and intentions are conveyed by an inter-modal fluency of action, voice, and touch (Stern et al., 1985; Trevarthen et al., 2011; Trevarthen and Delafield-Butt, 2013). And their timing and particular kinematic form transmit affective value, giving particular expressive, poetic feeling that holds meaning for those with whom they are shared (Stern, 2010). The timing, form, and energetic of body movements can be specific to a culture and learned in early adult–infant engagement (Gratier and Trevarthen, 2008; Gratier and Apter-Danon, 2009). Feelings conveyed in body movement, in choice or form of action, form a basis of cultural knowledge, and evolution (Rogoff, 2003; Hrdy, 2009; Packard and Delafield-Butt, 2014).

The present study examines the early development of cultural differences in communication between mothers and infants in ‘pick-up and carry’ paradigm by application of high-precision motion capture, together with video micro-analysis, to accurately record the actions of mother and infant during this task. Special attention is paid to the timing and structure of the mother’s movements as we reason it provides a culturally specific framework for the full sequence of the ‘approach,’ ‘pick-up,’ and ‘carry’ phases as a determinant of culture-specific behavioral development. Mother–infant interactions were examined by video micro-analysis of data obtained from cameras set alongside the motion capture system. Together, motion capture and video data afforded a comprehensive analysis of both kinematic style and quality of expressive behavior, giving precise measure to the inter-body relationship between mother and infant from two different cultures (Japan and Scotland) at two developmental ages (6 and 9 months).

### TEMPORAL COORDINATION IN INTERSUBJECTIVITY

Mothers are commonly understood as the principal driver of an interaction, structuring the encounter and framing it. However, infants are also active participants in social interaction evident from birth (Nagy, 2011), both soliciting interaction from others (Nagy and Molnar, 2004) and patterning these to form intersubjective dialogs of meaning-making (Trevarthen and Delafield-Butt, 2013; Kugiumutzakis and Trevarthen, 2015). A wealth of detailed mother–infant analyses prove that adjustment of the timing of actions that make up behavior is facilitated by awareness of each partner’s intention, and mother and infant read the intentions inherent in each other’s actions, coordinating their activity and expressions, and forming the basis of embodied intersubjectivity (Brazelton, 1979; Stern, 1985; Trevarthen, 2001).

Mother and infant communicate with each other to generate shared meaning. The communication has components of pulse, quality, and narrative with a four-part structure of introduction, development, climax, and resolution. Thus, mother–infant interaction generates what Malloch and Trevarthen (2009) identify as ‘*communicative musicality.*’ In this idea, synchrony is not only shared dynamically between individuals, but is also contextual. Such context-based interactions enable participants to dynamically anticipate each other’s behaviors and sequentially attune their own behaviors to them, such as in jazz improvisation (Schögler and Trevarthen, 2007). This kind of successive anticipation, intention-reading and resulting sequence of joint engagement promotes a sense of belongingness (Gratier and Apter-Danon, 2009). These interactions require a complex reciprocity in the behaviors between the mother and infant. Nine months of age is the time of joint attention and is interpreted as the time of significant development in intention-reading in a triadic relationship (Tomasello, 1993), but younger infants are nevertheless aware of the social context and adapt their actions appropriate to their particular feelings and motivations within it (Legerstee and Markova, 2007; Ishijima and Negayama, 2013).

Trevarthen (1998) identified two different types of intersubjectivity: primary intersubjectivity and secondary intersubjectivity. Primary intersubjectivity involves direct social attention and attunement evident from birth, while secondary intersubjectivity, characterized by inclusion of objects into the primary mother–infant intersubjective interactions, is evident from 9 months (Trevarthen and Hubley, 1978). Joint attention of mother and infant to an object of shared interest is considered to be a mutual inclusion of the other’s perspective into their shared experience to form a true triadic relationship (Tomasello et al., 1993). Mutual intention-reading between mother and infant enables advanced, fine temporal coordination between infant and mother at 9 months. However, mothers and infants take part in shared attention and engagement with their body parts in games and rituals, such as in tickling play, which suggests an earlier form of proximal triadic relations using their body part as the target may exist (proto-triadic relation; Negayama, 2011).

Another important concept closely related to intersubjectivity is parent–infant interactional synchrony; synchrony requires mutually adaptive timing. Feldman (2007) identified synchrony as a construct that denotes intersubjectivity. Synchrony has several different developmental phases, starting with a basic biological clock and autonomic physiological system, through to voluntary, behaviorally mediated interactions and symbol use. Among such interactions, touch is a strong inducer of synchrony. Touch is a significant modality of agent engagement with strong, direct sensory consequences that can be life-affirming, or the opposite. It simultaneously brings a bilateral experience of ‘touch’ and ‘being touched’ in the participants (Rochat, 2001), and this bilaterality of experience is a significant mediator of synchrony. Experience of contact is always mutual, and the experience is intensely personal; it is not shared by a third other and it generates vital, affective appraisals of their value as benefit or threat.

For example, hugging and kissing mediate affection, but hitting and kicking are aggressive attacks. Thus contact can elicit broad range of emotions. And as the body is isomorphic between the persons mutually engaging touch, the sensations of one’s body being touched is simultaneously sympathetically perceptive to the one making the touch. Such unique characteristics of symmetry and simultaneity in tactile experience are favorable for conveying shared feelings of oneness between mother and infant. Touch is not simply a tactile experience of texture and pressure, but involves different types of receptors all over the body, including those for temperature and pain (McGlone and Spence, 2010). Bodily communication with touch gives a rich and intimate experience to both participants, of which holding and being carried is one important everyday example.

### HOLDING BEHAVIOR AND ITS DEVELOPMENT

Opportunities to learn and practice synchronization of one’s own behavior to another’s behavior are richly embedded in everyday-life tactile interactions. Mother–infant holding is a behavior of this kind because it is a major joint behavior of the mother and infant that requires fine tactile attunement of movements in the arms, hands, legs, and trunk (Negayama et al., 2010). Holding behavior is clinically known to reflect the quality of the mother–infant relationship (Massie, 1975; Weatherill et al., 2004) possibly because of the necessity of intimacy for this complex mother–infant coordination.

Hand-aiming, clutching and lifting by the mother, and arm-reaching and grabbing by the infant are likely to be included in the paradigmatic sequence of ‘put-down’ and ‘pick-up’ phases. When the mother walks, she and her infant dynamically adjust their behaviors in harmony with each other to maintain secure holding. For all these to be performed smoothly, the mother and infant must mutually attune their movements precisely.

The ‘put-down’ phase is a separation of the infant, previously securely held by his or her mother, from the mother, and the ‘pick-up’ phase is thus a reunion with the mother who just left the infant alone. However, the mother–infant interactions within these processes have the opportunity to be even more dynamic than during the simple act of holding, and mother–infant adjustment of behaviors for synchrony and reciprocity should be worth examining in the paradigmatic sequence of put-down, pick-up, and carry.

Reddy et al. (2013) demonstrated that even infants of 2 months of age are able to adjust their behaviors in anticipation and in preparation of being picked up. This responsiveness develops over the following few months. As noted above, 9 months of age marks an upsurge in intention-reading. Thus, the interactions during the pick-up phase ought to be different between 9 months old and younger ages. Successful pick-up requires a precise attunement of timing in embodied communication, which might require a long developmental and learning process that precedes the 9-months revolution and transition to true secondary intersubjectivity.

### CULTURAL DIFFERENCE IN PARENTING

Two types of parenting have been repeatedly pointed out as a cultural difference: regulator or authoritarian type and facilitator or authoritative type. Japanese parenting is classified in the latter (facilitator) type, which relies more on affective ties and empathy rather than parental control. In the authoritative childcare in Japan, children are expected to take the parent’s intention on their own and control their behavior accordingly. In addition, Japanese mothers are characterized by their tendency to follow, not control, the infant (Azuma, 1994).

Keller et al. (2009) proposed another dichotomy in parenting types: distal and proximal. Japanese mothers and infants engage in more bodily contact than their U.S. counterparts (Rothbaum et al., 2000), and can be classified into the proximal type. Japanese parents feel less averse to their infants’ bodily waste than French parents do (Negayama and Norimatsu, 2009), which is supportive evidence of a stronger psychological closeness to the infant body in Japanese culture. As mentioned previously, bodily contact brings a feeling of oneness, and the Japanese authoritative parenting relies on this mutually minded attunement even when physically separate.

Japan and Scotland are also culturally different in the structure of childcare observed in, e.g., behaviors of feeding (Negayama,1998–1999) and of the caregiver–infant relationship in putting children to bed (Negayama and Kawahara, 2010) in day nurseries. These studies showed a stronger Japanese motivation to comfort the infants patiently and with greater contact. This may be reflected in the process of pick-up and holding studied in the present paper. Mother-infant attachment patterns have been classified into four types (secure, avoidant, ambivalent, and disorganized) by the Standardized Strange Situation paradigm (Solomon and George, 2008). The paradigm also shows a remarkable cultural difference (IJzendoorn and Sagi-Schwartz, 2008).

All these findings are related to cultural differences in mother–infant intersubjectivity, and we expect these differences could result in differences of timing and organization of behavioral patterns observed in the put-down, pick-up, and carry paradigm. Thus, fine kinematic analysis of interaction during these phases is likely to be a sensitive and promising window with which to explore the development of these cultural differences in the mother–infant relationship.

### SUMMARY OF THE AIM

The aim of this paper is to identify and define culturally and developmentally specific patterns of motor timing and form by kinematic analysis of maternal movements and mother–infant behaviors. Mother–infant pairs in Japan and Scotland were observed once at 6 months and once at 9 months. An identical procedure was employed at both sites and at both ages to afford comparison of action and interaction timing and forms before the onset of secondary intersubjectivity and at its onset (Trevarthen and Hubley, 1978) at the so-called 9-months revolution (Tomasello, 1995).

## MATERIALS AND METHODS

### PARTICIPANTS

Eleven Japanese and ten Scottish healthy infants between the age of 5 and 6 months participated with their mothers. They were recruited at local nursery schools in Japan and through word of mouth, parent groups, and nurseries in Scotland. Mother and infant pairs participated in the experiment twice: first at the infants’ age of ca. 6 months, and second at ca. 9 months. This study is a part of a bigger project on the development of mother–infant gap closure with three different approaches of picking up, feeding, and playing. Six months was chosen as the normal starting age of solid-food. Background information of the participants is shown in **Table 1**. This study was approved by the Ethical Review Board of Waseda University (No. 2012-273). Written informed consent was obtained from each mother or father.

**Table 1:** Japanese and Scottish mothers and infants participated in the study.

Country	Dyads	Infant’s birth order	Infant’s birthdate	Sex	Age in days at first recording	Age in days at second recording	Mother’s age in years	Mother’s final education	Mother’s occupation
Japan	J1	2	2011/9/2	Girl	185	292	32	Vocational college	Full time
	J2	2	2011/9/14	Girl	180	285	33	University	Full time
	J3	2	2011/9/22	Girl	192	295	39	Junior college	Housewife
	J4	1	2011/9/17	Boy	203	283	29	Vocational college	Housewife
	J5	2	2011/9/22	Girl	202	299	29	Junior college	Part-time
	J6	1	2011/10/20	Girl	185	288	29	University	Housewife
	J7	3	2011/11/6	Girl	192	281	39	Junior college	Housewife
	J8	2	2011/11/20	Girl	183	269	33	University	Full time
	J9	1	2011/11/8	Boy	197	299	35	University	Full time
	J10	1	2011/11/13	Girl	207	268	31	Junior college	Housewife
	J11	1	2011/12/6	Girl	186	245	31	University	Full time
	
	**Average**				**192.0**	**282.2**	**32.7**		

Scotland	S1	1	2012/2/8	Girl	209	293	30	Graduate school	Housewife
	S2	3	2012/5/9	Boy	187	285	39	Junior high school	Part-time
	S3	1	2012/5/31	Girl	179	298	37	University	Full time
	S4	2	2012/8/18	Boy	223	289	30	Junior college	Self-employed
	S5	2	2012/5/19	Boy	195	299	35	Senior high school	Full time
	S6	1	2012/5/5	Girl	208	324	28	University	Full time
	S7	1	2012/6/27	Boy	156	281	21	Junior college	Full time
	S8	1	2012/5/3	Girl	211	293	41	Junior college	Full time
	S9	1	2012/7/8	Boy	156	276	29	Junior high school	Full time
	S10	1	2012/3/14	Girl	**–**	260	35	Graduate school	Full time
	
	**Average**				**191.4**	**289.8**	**32.5**		

### PROCEDURE AND DATA RECORDING

Data recordings of Japanese and Scottish participants were carried out at a laboratory at Waseda University and another at the University of Strathclyde, respectively. Participants’ visit schedules were tailored to fit the infants’ eating and sleeping patterns so the infants arrived in an awake and alert phase some 30 min prior to typical feeding time.

All mother and infant pairs performed pick-up and carrying tasks at 6 and 9 months. This study is a part of the bigger research project, and the present task was performed at first of four different tasks: (i) mothers put-down, then picked up their infant from the floor, (ii) mothers fed their infant with solid-food with a spoon, (iii) mothers tickled their infant in free play of about 15 min, and (iv) mothers and infants played an action-word game task. During the tasks, mothers’ and infants’ body movements and interactions were audio–video recorded with two or three standard consumer digital video cameras and their movements recorded by optical motion capture systems. Each digital video camera was either mounted on a tripod to record the mother’s and infant’s whole body within the frame, or hand-held to allow focus on eye gaze and specific facial expressions.

For motion capturing, comparable 3D motion analysis systems were employed: a 12-camera Optitrack system (NaturalPoint Inc., USA) employed at Waseda University in Japan, and a 12-camera Vicon Nexus system (Oxford, UK) employed at the University of Strathclyde in Scotland. Reflective markers were attached to the mother’s head, shoulder, back, arm, hand, and waist, and infant’s head using a similar configuration. In Scottish cases, additional markers were attached to the infant’s shoulder, back, arm, hand, waist, and leg. Optical motion capture data were collected at 100 Hz. The motion capture floor space was marked by use of nearly identical, soft brown carpets measuring 2600 mm × 2000 mm and 2300 mm × 3300 mm for the Japanese and Scottish situations, respectively. The room itself was larger in Scotland and facilitated greater freedom of movement. The room floor sizes were ca. 3.9 m × 9.9 m with a height of 3.0 m in Japan and ca. 8.0 m × 18.0 m with a height of ca. 4.0 m in Scotland.

After finishing all tasks, information on mothers’ and infants’ birth date, sibling number and parity, maternal education and employment, and health were collected by brief interview (see **Table 1**).

### MOTION ANALYSIS

**FIGURE 1:**
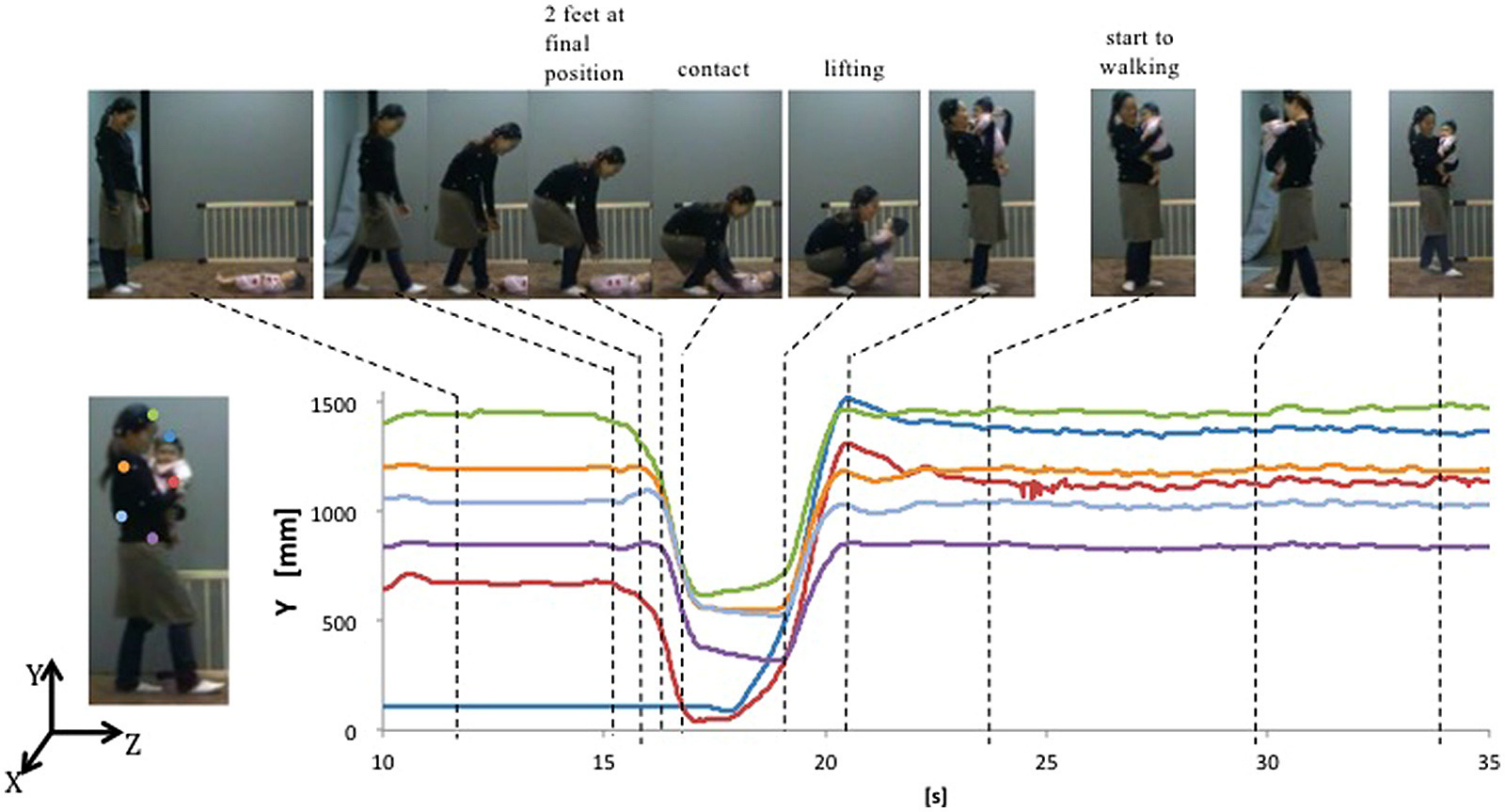
**A Japanese mothers movement and body’s trajectories during pick-up and holding (approaching phase).** Part of annotated typical behaviors are shown at the top.

Each mother’s and infant’s motion during the ‘approaching’ phase of the pick-up and carrying task was focused on and analyzed to better understand the intersubjective engagement (**Figure 1**). Body part trajectories were obtained either from a calculation of the average position of three or four markers placed on a rigid surface and attached to each body part (Japan), or from single marker displacements placed directly on the body (Scotland).

Each trajectory was time-shifted to *t* = 0 at the mother’s contact point with her infant. The contact point was determined by calculation of 5% of the maximum velocity of the distance-gap closure between mother’s hand and infant’s head in the following steps: (i) calculating the distance sequence between mother’s dominant hand and infant’s head positions, (ii) velocity sequence was obtained by calculating differences between adjacent values of the distance-gap trajectory, (iii) velocity trajectory was smoothed by Gaussian smooth function whose cut-off frequency was 9 Hz, (iv) two 5% of maximum velocity points were obtained for each approach, and (v) the second 5% point was determined to be the contact point. This contact point was used to assess the mothers’ and infants’ coordination of body movement, kinematics, behavioral and affective expressivity, and motor anticipations during the mothers’ approach to pick-up her child.

In this approaching phase, mother’s typical motions were annotated by observation of the video. The time-points at which (i) the mother stopped walking (two feet at final position), (ii) touched her infant’s body (contact), (iii) lifted up infant’s entire body from the floor (lifting), (iv) finished picking up and started to hold the infant in a stable manner (stable holding), and (v) started to walk by lifting the foot (walking), were chosen. These time course was analyzed together with motion-captured data.

One trial was selected for each pair at 6 and 9 months on the basis of the infants’ emotional stability and the behavioral visibility. We selected an earlier trial if more than one trials met the standard. Then 11 Japanese and 10 Scottish mothers’ trajectories were evaluated, and their kinematic parameters were compared in each 6- and 9-months group.

### BEHAVIORAL ANALYSIS

Based on an idea that the current put-down/pick-up procedure is regarded as a simplified separation-reunion situation, “leaving” and “approaching” phases were annotated in ELAN (Max-Planck Institute for Psycholinguistics, Nijmegen, The Netherlands). The “leaving” phase was defined as starting with the mother’s placement of her infant on a floor and ending with the stop of her stepping back. The “approaching” phase was defined as section from 2.5 to 0.5 s before touch.

Four infant behaviors were coded for each phase: eye gaze, negative reaction, positive reaction, and arm reaching. Occurrence of eye gaze was judged by the visual or facial orientation to mother irrespective of the frequency or intensity. Negative and positive reactions were coded on the basis of facial expression, tone of voice, and body movement. Occurrence of arm reaching was judged by the extension of arm to mother irrespective of the frequency or intensity. Inter-rater reliability between two experienced coders was calculated by independently coding all the Japanese data, and was within an acceptable range (Kappa was 0.75). Kappa’s for eye gaze toward mother, negative reaction, positive reaction, and arm reaching calculated by independently coding randomly chosen 30% of data were 0.92, 0.89, 0.67, and 0.91, respectively.

Further, the greeting-like behaviors in mothers (hand/arm opening and vocalization) and infants (leg flailing and vocalization) were annotated and timing of the occurrences was measured by ELAN. Mothers’ hand/arm opening was a quick extension of fingers and/or arms just before pick-up, and infants’ leg flailing was a jerky movement of lifted legs. Inter-rater reliability for each behavior was calculated by coding 14 randomly chosen pairs by a second experienced coder, and was within an acceptable range: Kappa’s for mother’s hand/arm opening and vocalization and infant’s leg flailing, vocalization were 0.96, 0.81, 0.92, and 0.85, respectively. Then a gap between the mother’s hand and the infant’s head at the moment of the behavior occurrences was calculated from the motion capture coordinate values of the markers.

Holding is a joint behavior requiring active participation between mother and infant after the infant acquires motor control (Negayama et al., 2010). The style of maternal holding was analyzed by recording the placement of the mothers’ hand holds on her infant, which fell into two categories: infant’s back/armpit and bottom. Maternal 2-hand positions were classified into two patterns: bottom-back/armpit and bottom–bottom. The latter pattern requires autonomous posture control by the infant and allows the infant’s free body movement, both signs of advanced development. Kappa for the judgment of maternal hand position was 0.72.

## RESULTS

### ACTION PATTERNS AND KINEMATICS OF MOTHERS’ APPROACH

Picking up the infant from the floor required transport of the hand to the grasping point of the infant along the trunk and under the arms. In this paradigm, mothers began their approach several paces away from their child and therefore control of gait, leaning, and/or kneeling or squatting was required to move into proximity of the child, altogether enabling displacement of the hands to the point of best purchase along the infant’s trunk.

**FIGURE 2:**
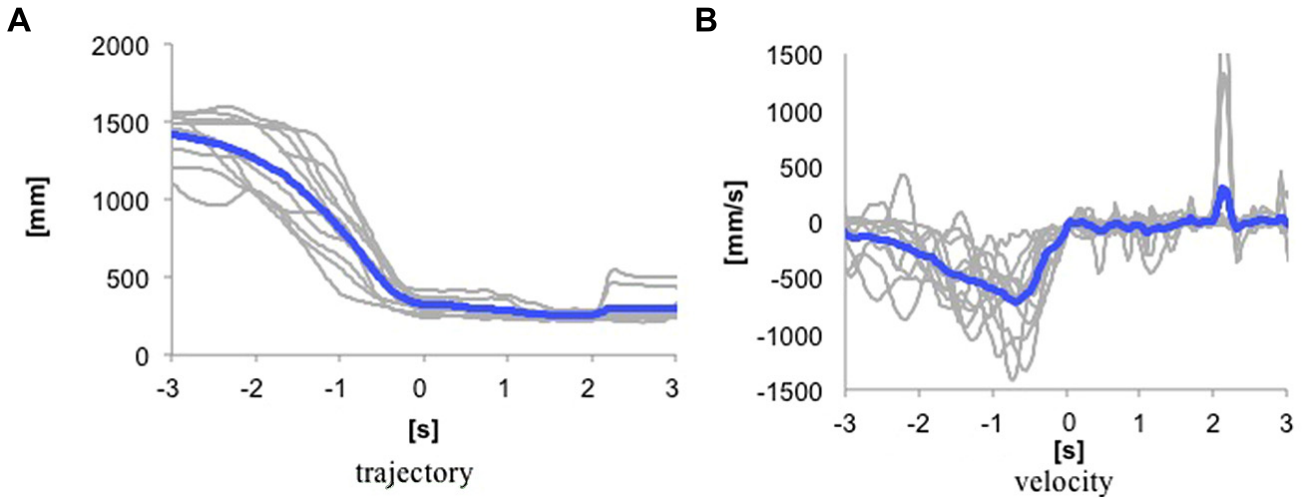
**Examples of closure of Japanese mothers’ hand to their infants on approach to pick-up.** [**(A)** trajectory of mothers’ hand height, and **(B)** velocity of mothers’ hand movement.] Mean distance between hand of mothers and head of infants at 6 months are given in **bold.** Individual gap closures are given in gray. The contact point, *t* = 0, is calculated as 5% of the maximum velocity of the gap closure.

Trajectories of distance between mother’s dominant hand and the infant’s head in each of mother–infant dyads at 6 months are given in **Figure 2A**. The curves are very smooth, which means that the approach was generally constant. In spite of the smooth approach, the speed of movement in the mothers’ dominant hand to reach the infants was highly variable as shown in **Figure 2B**. The gap closure of the approach became smooth due to this moment-by-moment adjustment of velocity of the hand to absorb different speeds of movement in different body parts to reach its goal efficiently.

**FIGURE 3:**
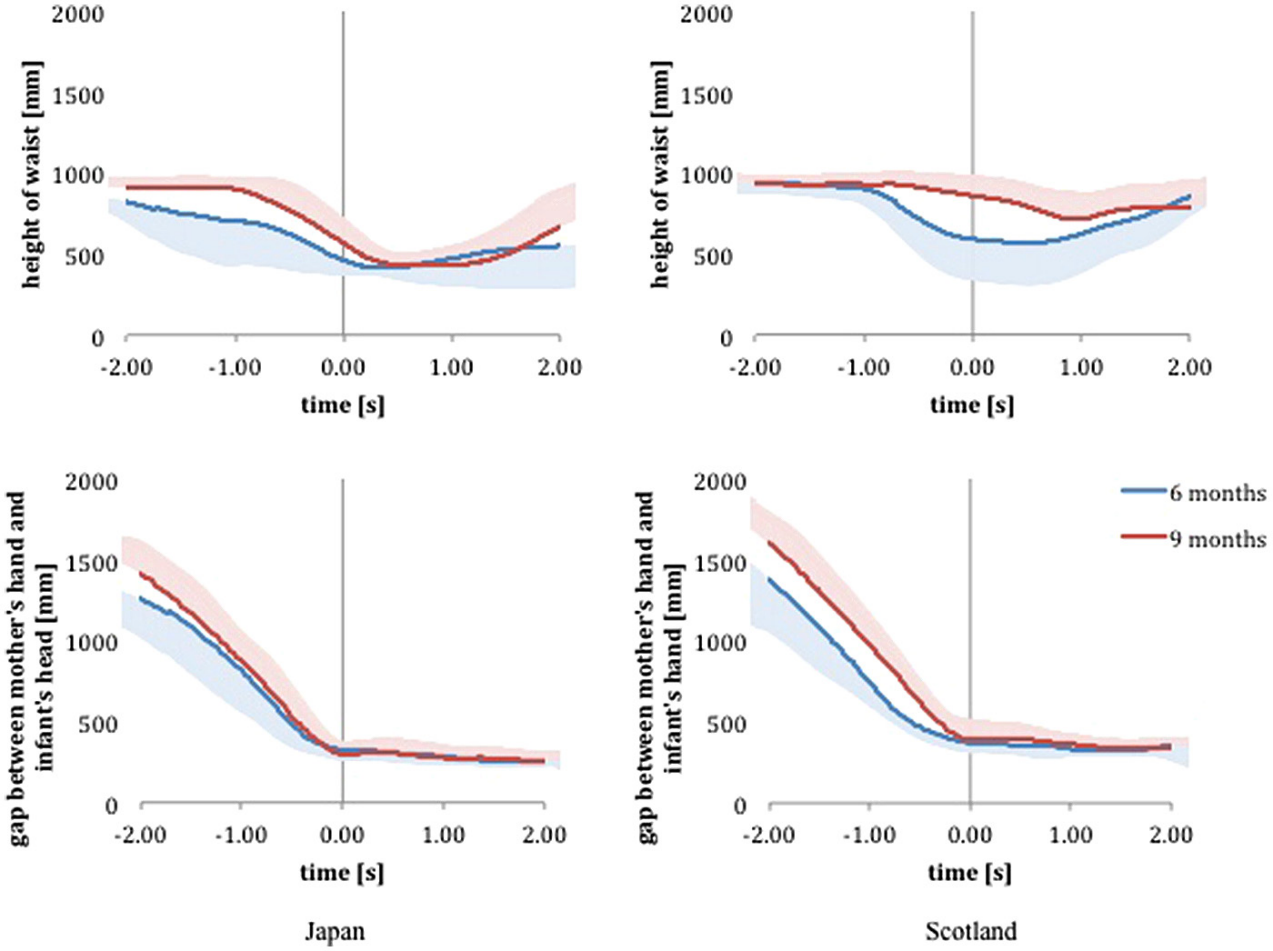
**Mean and standard deviation of height of mothers’ waist (top), and distance between mother’s hand and infant’s head (bottom) during the approach and pick-up.** Lower and upper standard deviation only are given for 6 and 9 months, respectively, to preserve clarity. Note the shorter distance and greater variation in Japanese gaps at circa 2 s prior to contact at 6 months, but not at 9 months. By 9 months these differences have disappeared and the two populations are more comparable. The contact point, *t* = 0, was calculated as 5% of the maximum velocity of the mother–infant gap closure.

Japanese and Scottish mothers approached their infants with different styles. Changes in the height of waist in **Figure 3** show that the Japanese mothers stepped forward with crouching or kneeling at the feet of their infants than the Scottish mothers at 6 months, giving earlier closeness in proximity during the approach. Duration of squatting at 6 months was significantly correlated with age (days) of the infants in the Japanese pairs (i.e., longer squatting for the younger infants, Pearson’s *r* = –0.633, *p* = 0.037). On the other hand, the Scottish mothers were characterized by a higher waist position at 9 months than the Japanese mothers and by the lack of kneeling and squatting, with the exception of a one-knee kneel by one mother with difficulty in picking up her infant. The Scottish mothers picked up their infant by just bending the torso forward.

**FIGURE 4:**
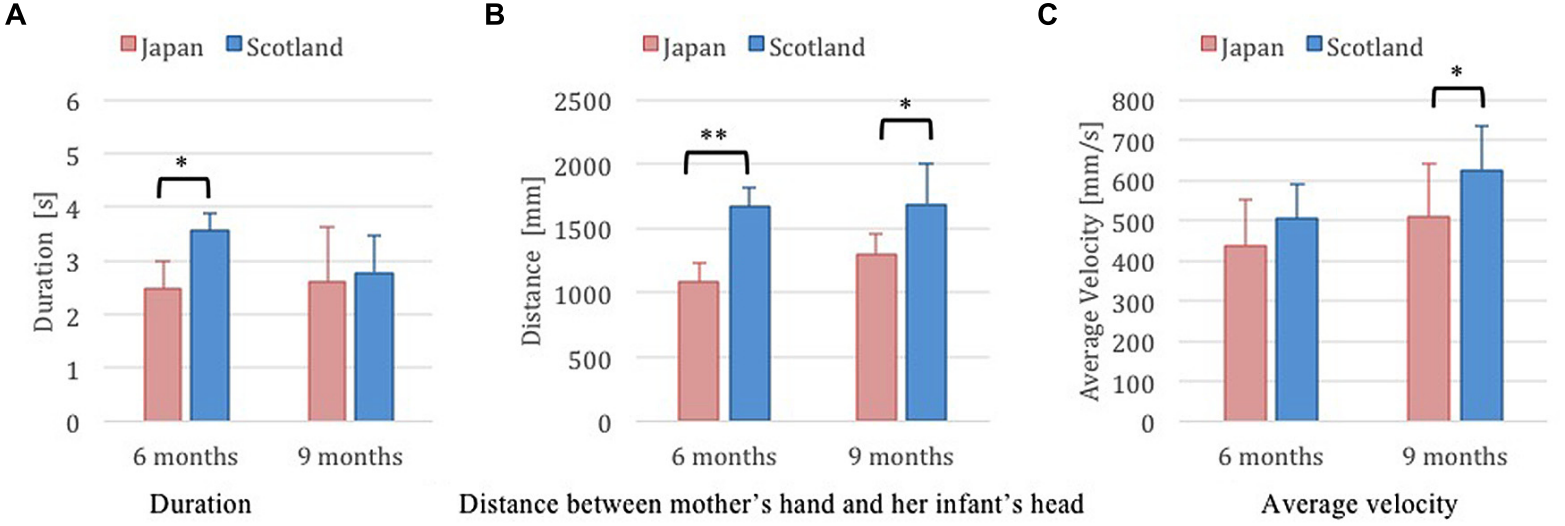
Kinematics of the final continuous gap closure between mother’s hand and her infant [**(A)** duration of the final continuous closures, **(B)** distances of the closure, **(C)** average velocity of the closure; *p < 0.05, **p < 0.01]. Discontinuous movements were excluded.

We reasoned the kinematics of the continuous gap closure between the mother’s hand and the head of the infant on approach to contact (**Figure 4**) was a good marker of the quality of the approach, indicated by computations of duration, velocity, and distance of approach. Of the approaches analyzed, some movements exhibited discontinuous gap closures due to long pauses in the kneel/squat phase in the case of Japanese mothers (one at 6 months, three at 9 months), or clapping and arm waving in the case of Scottish mothers (one at 6 months, two at 9 months) and were excluded. The remaining movements were comparable in producing a single continuous velocity to contact with the infant.

Analyses of variance (Welch’s test) of these continuous closures of the hand to the point of contact with the child (**Figure 4**) revealed significant differences in kinematics between Japanese and Scottish mothers’ movements at 6 months and at 9 months. The duration of the final continuous closures was significantly longer in Scottish mother–infant pairs than in Japanese pairs at 6 months, but not at 9 months (**Figure 4A**). The distances of the closure was significantly longer in Scottish pairs than in Japanese pairs at both 6 and at 9 months (**Figure 4B**). Finally, the average velocity of Scottish mothers at 9 months was significantly greater than that of their Japanese counterparts (**Figure 4C**), but this was not the case at 6 months.

### SEQUENTIAL TIMING OF PICK-UP BEHAVIORS

The sequence of movements made by mothers to pick-up their infants was mapped to produce a sequence of time-points: starting with two feet reaching the final position before contact, contact with the infant next, then onset of lift, and finally onset of walking (i.e., carrying). Correlation analysis of these time-points among the mothers at each age revealed a higher correlation between behaviors at 6 months for both Japanese and Scottish mother–infant pairs than at 9 months (**Table 2**). Further, Japanese mothers at 6 months demonstrated stronger correlations with more behaviors correlated than their Scottish counterparts, indicating more regular, structured coordination in their sequence of actions with greater similarity among the mothers. Interestingly among the correlated behaviors, there was no significant correlation between timing of final foot position and contact in Scottish mothers, whereas timing of this final foot position was significantly correlated with those of contact and lift, and timing of contact was significantly correlated with those of all other behavioral markers for the Japanese pairs. This suggests a sequential programmed engagement that purposefully accounted for the foot position in Japanese, but not in Scottish pairs, which suggests a more various and flexible patterning in Scottish mothers. Significant correlations were seldom observed at 9 months for both populations.

**Table 2:** Temporal organization of mothers’ apprach, pick-up, and put-down.

		6 months				9 months			
		Contact	Lifting	Walking	Stable holding	Contact	Lifting	Walking	Stable holding
Japan	2 feet at final position	0.777**	0.611*	0.55	0.513	0.31	–0.168	0.272	0.099
	Contact		0.814**	0.787**	0.780**		0.594	0.772**	0.588
	Lifting			0 964**	0.917**			0.469	0.183
	Walking				0 984**				0.602
									
Scotland	2 feet at final position	–0.249	–0.386	–0.514	–0 787*	0.699*	0.345	–0.187	0.087
	Contact		0.557	0.569	0.481		0.478	–0.269	–0.048
	Lifting			0.754*	0.771*			0.011	0.246
	Walking				0.834*				0.787**

Pearson’s r between onset times of sequential pick-up behaviors for Japanese and Scottish mother–infant pairs at 6 and 9 months (*p < 0.05, **p < 0.01).

### CONTACT THROUGH EYE GAZE, VOCAL, AND GESTURAL COMMUNICATION

Japanese and Scottish infants differed markedly in the amount of expressive gestural action and vocalization made during their mothers’ approach to pick-up, with Scottish infants more active and expressive than the Japanese ones (**Table 3**). The paradigm involves the mother placing the infant on the floor and withdrawing a few steps before approaching again, presenting a mild separation and reunion between mother and infant.

**Table 3:** Incidence of infants’ expressiveness and sensory contact between mother and infant during withdrawal and approach.

		6 months						9 months					
		Withdrawal			Approach			Withdrawal			Approach		
		Japan	Scotland	*p*^a^	Japan	Scotland	*p*^a^	Japan	Scotland	*p*^a^	Japan	Scotland	*p*^a^
Eye gaze toward mother	Occurred	4	8	0.007	6	8	0.040	6	9	0.094	7	9	0.185
	Not occurred	7	0	5	0		5	1		4	1	

Negative reaction	Occurred	2	3	0.336	1	1	0.678	4	4	0.608	3	2	0.5
	Not occurred	9	5		10	7		7	6		7	8	

Positive reaction	Occurred	0	0	–	2	4	0.166	0	1	0.476	2	4	0.314
	Not occurred	11	8		9	4		11	9		8	6	

Arm reaching	Occurred	1	1	0.678	0	4	0.018	1	0	0.524	4	6	0.26
	Not occurred	10	7		11	4		10	10		7	4	

*^*a*^Fisher’s exact test.*

The difference between Scottish and Japanese was checked by Fisher’s exact test. Scottish infants looked at their mothers more often than Japanese infants did. At both 6 and 9 months, most Scottish infants (8/8 and 9/10, respectively) maintained eye gaze with their mothers as they withdrew, while a smaller proportion of Japanese infants did (4/11 and 6/11; *p* = 0.007 and 0.094, respectively). And at 6 months all Scottish infants (8/8) held their gaze on their mothers as they approached, but only about half of the Japanese infants did (6/11; *p* = 0.040). Scottish infants reached with their arms and hands more often than Japanese infants did at the approach phase at 6 months (0/11 and 4/8 for Japanese and Scottish, respectively, *p* = 0.018), but at 9 months no significant difference was observed (4/11 and 6/10 for Japanese and Scottish, respectively; *p* = 0.260).

Mother and infant ‘greeting’ behaviors in the approach phase differed between cultures (**Table 4**). Scottish infants vocalized at approach at 6 months, but the Japanese infants did not (1/11 and 6/8 for Japanese and Scottish, respectively, *p* = 0.006). Further, at 9 months, a greater proportion of Scottish infants tended to flail their legs as their mother approached than did the Japanese infants (1/11 and 5/10 for Japanese and Scottish, respectively, *p* = 0.055). On the mothers’ side, there was also some tendency that a greater proportion of Scottish mothers showed greeting-like arm and hand gestures at 9 months than Japanese mothers did (2/11 and 6/10 for Japanese and Scottish, respectively, *p* = 0.063), although both Scottish and Japanese mothers were unlikely to display it at 6 months (1/11 and 3/8 for Japanese and Scottish, respectively). Finally, there appeared to be balance between the proportion of mothers who vocalized on approach to their child between populations and ages.

**Table 4:** Mothers’ and infants’ ‘greeting’ behavior incidence in the approach phase for reunion.

		6 months			9 months		
		Japan	Scotland	*p*^a^	Japan	Scotland	*p*^a^
Mother’s arm/hand opening	Occurred	1	3	0.177	2	6	0.063
	Not occurred	10	5		9	4	

Mother’s vocalization	Occurred	7	6	0.494	6	6	0.575
	Not occurred	4	2		5	4	

Infant’s leg flailing	Occurred	3	4	0.297	1	5	0.055
	Not occurred	8	4		10	5	

Infant’s vocalization	Occurred	1	6	0.006	3	5	0.268
	Not occurred	10	2		8	5	

*^*a*^Fisher’s exact test.*

Altogether, it appears the Scottish infants monitored their mothers more carefully and were motivated to react to the situation more actively than Japanese infants at 6 months, Scottish mothers also tended to show more frequent bodily gesture. However, there were no significant differences in the positive or negative expression of affect *per se*, which means that although Scottish mothers and their infants produced and maintained more overt and direct sensory contact, their affective experiences did not appear to be dissimilar.

### INFANT AND MOTHER INTIMATE SPACE

**FIGURE 5:**
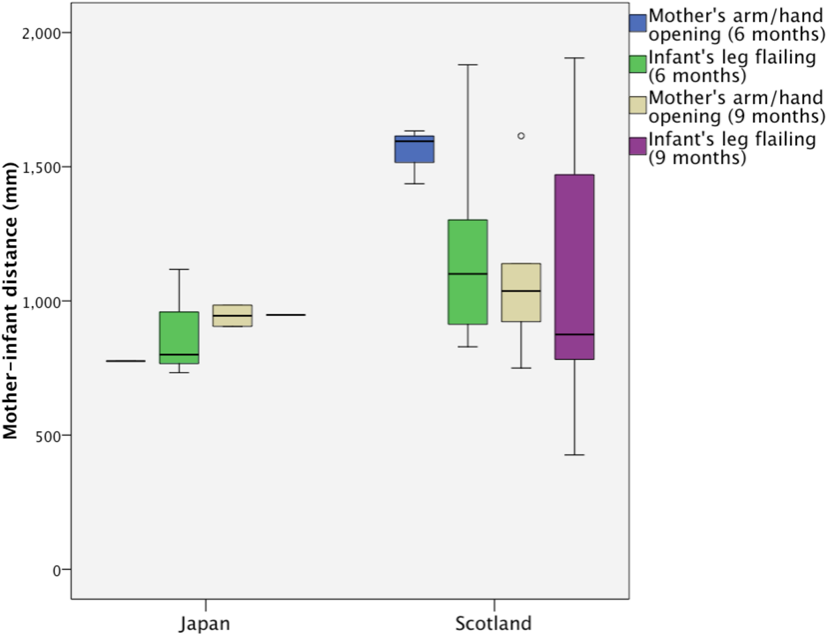
**Box plot of distance between the mother’s hand and the infant’s head at the moment of the greeting-like behaviors of mother’s arm/hand opening and infant’s leg flailing at 6 and 9 months for Japanese and Scottish pairs.** These behaviors were shown at a narrow distance around 1 m for both countries, suggesting a peri-personal space in them.

The distance between mother’s hand and infant’s head at the moment of expression of particular styles of greeting was measured between the locations of markers on the mother’s hand and infant’s head. Expressions of greetings from both mother and infant occurred in a zone between 400 and 1,800 mm with a stable median around 1 m from the infant’s head to the mother’s hand as the mother approached (**Figure 5**).

Median (and Quartile Deviation) of the gaps in all the greeting behaviors by mothers and infants at 6 and 9 months were 787 (57) mm and 948 (20) mm for the Japanese pairs and 1302 (288) mm and 1139 (515) mm for the Scottish pairs, respectively. The similarity at 9 months in spite of difference in experimental floor space between Japan and Scotland, suggests a common border at about 1 m between the mother’s hand and the infant’s head separating the intimate and outer spaces active in both mother and infant at 9 months.

Finally, the timing of occurrence of these behaviors was compared between mother and infant within each pair to determine if one or the other initiated expressive participation. The analysis failed to find any consistent initiator–follower relationship between them in either of the two ages (*p*’s = 1.00 and 1.00 for 6 and 9 months, respectively, by binominal test applied to greeting behaviors of mother and infants).

### MATERNAL HOLDING AND INFANT PARTICIPATION

The infant’s autonomous orientation was enabled by a change in the mother’s holding style. At 6 months, mothers predominantly held their infants with one hand on the infant’s bottom and the other hand on the infant’s back or armpit (73% of Japanese pairs and 88% of Scottish pairs), rather than using both hands to support the bottom. At 9 months, only about half of the mothers held their infants with one hand on the infant’s bottom and the other hand on the back or armpit (55% of Japanese pairs and 60% of Scottish pairs). The other half used both hands to support the bottom showing a more advanced style.

## DISCUSSION

Complex mother–infant interactions during the phases of approaching, picking up, and holding the infant were analyzed at two developmental ages, 6 months and 9 months, which correspond to the period immediately preceding and immediately after the onset of secondary intersubjectivity and joint attention. Motion analysis together with video micro-analysis revealed interesting age and cultural differences.

### MOTHER–INFANT MOTION ATTUNEMENT IN PICK-UP AND HOLDING

The smooth approach of the mothers’ hands was produced by moment-by-moment adjustment of its speed (Bernstein, 1967). The degree of smoothness of the hand movement as it approached may affect the infant’s anticipation and adaptive motor response. Reddy et al. (2013) found that infants as young as 2 months prepared to be picked up by their mothers with postural adjustments to muscle tensions in the back, neck, hands and legs, as well as in expressive gestural communication. This increase in muscular strength and regulatory autonomy appears to develop a behavioral-biological pattern that may have facilitated more autonomous infant attunement with their mother’s behavior, enabling a common, shared goal orientation. These behaviors also give some indication for understanding the more efficient pick-up at 9 months as a more active, joint collaboration between mother and infant than at 6 months.

Holding is a complex joint action between mother and infant (Negayama et al., 2010), and is also part of wider context including the approach before pick-up. Mothers and infants of less than 1-year-old cooperated to make a smooth pick-up possible. Our data suggest that early onset of social action anticipation continues to develop and improve over the first year of life, and is made in a collaborative fashion by both mother and infant to actualize culturally different fine attunements for efficient and smooth pick-up.

Ishijima and Negayama (2013) observed Japanese mother–infant tickling interaction longitudinally and found an expectant ticklishness in infants before an actual touch by the mother at 6 ½ months of age. Such anticipation of contact in everyday-life interactions (e.g., tickling or holding) is a sign of awareness of the other’s intention. Such awareness may support social cognitive development as it progresses from primary intersubjectivity to secondary intersubjectivity, developing ‘mind-reading’ of the other’s intentions in anticipation of its action consequence (Sinigaglia and Rizzolatti, 2011), especially during regularly patterned episodes of inter-body interaction (Delafield-Butt and Trevarthen, 2013; Trevarthen and Delafield-Butt, 2013).

A disturbance in the organization of action at 9 months in both countries, on the other hand, was possibly caused by greater initiative and participation of the infants at this age that demanded compensations and adjustments from the mother. Infants were lighter and required less strength at 6 months, potentially freeing up one hand for additional support. Improved postural control by the infant and self-regulated stability of the upper body at 9 months may have allowed the mothers to use both hands to support their bottom, allowing for their increased weight to be supported safely with both hands. It also allowed more freedom for the 9-month-olds to turn and face the same direction as the mothers to share the perspective while walking.

### CULTURAL DIFFERENCES IN MOTHER–INFANT INTERACTIONS AT PUT-DOWN AND PICK-UP

The paradigm of the present study could be taken as a milder version of the separation-reunion situation the Strange Situation paradigm employs. Researches using the Strange Situation indicate that Japanese infants protest at separation (Ujiie and Miyake, 1985; Takahashi, 1990). But Takahashi (1990) reported a reduction in the distress response of Japanese children in a more familiar and typical situation than the standardized Strange Situation. Separation in the present study, with the mother kept within sight and within a few steps of the infant, further ameliorated the distress of separation for the Japanese infants, who were calmer than their Scottish counterparts.

Scottish and Japanese mothers showed different ways to actualize embodied intersubjectivity with different reactions to the mild separation and reunion on the basis of different cultural frameworks of mother–infant relationship. The measured response in the reunion phase somewhat parallels the Strange Situation (Ainsworth et al., 1978), which includes a measure of how an infant deals with separation anxiety by measurement of the affective expression and behavior in the reunion phase. Affect is not always expressed. Thus, we are interested in the reunion phase as indicative of mild separation affectivity, and how a culture negotiates these everyday feelings of separation and reunion. In another study, Japanese mothers typically stayed with their infants when putting them to bed, until they fell asleep. They were reluctant to leave them alone. This was in contrast to Scottish mothers who more often left their infants alone to fall sleep, even when crying (Negayama, 1997). Such cultural differences in the negotiation of separation may produce generalized, lasting differences in affective expectations in the to and fro dynamic of social relations.

In this study, Scottish mothers and infants explicitly tried to interact with each other, whereas Japanese mothers and infants were much less active. Almost all the Scottish infants looked at their mothers before being picked up, while Japanese infants seldom did so. A greater proportion of the Scottish infants reached their arms toward their approaching mothers and vocalized than did their Japanese infant counterparts at 6 months. These results suggest that the Scottish infants monitored their mothers carefully and anticipated the timing of contact, tried to interact with their mothers, and then reached their arms to cooperate with their mothers for the pick-up, especially at 6 months. Such expectation, cooperation and behavioral adjustment at 6 months in Scotland – before the so-called 9-months “miracle” or “revolution” (Tomasello, 1995) – is evidence of active anticipation of the patterns of participation made in regular patterns of embodied intersubjective engagement. This feature of social knowledge and awareness is clearly evident even at 2 months of age (Reddy et al., 2013), and data indicate it is active even at birth (Nagy and Molnar, 2004; Nagy, 2011; Kugiumutzakis and Trevarthen, 2015), with a first rudimentary social awareness emerging in mid-gestation fetal life (Castiello et al., 2010). The fact that Japanese infants exhibited less active expressivity at 6 and 9 months raises important questions on the cultural nature of social anticipation and its communicated affective expression – features that underpin attachment style classification. It is possible the Japanese infants were unaware or disinterested in the social patterns of engagement, but given the studies cited above we find this unlikely. Rather, it appears Japanese infants hold their social expectations differently, with different impulse for sharing expressively their affectivity.

A certain number of Scottish mothers took a higher waist position without squatting at contact. It resulted in the production of a greater mother–infant inter-body gap significant at both 6 and 9 months (**Figure 4B**). Scottish caregivers and infants are interpreted as being more distal than proximal (Keller et al., 2009; Negayama and Kawahara, 2010), and some Scottish mothers’ higher waist position might be a reflection of, or contribution to, this greater inter-body distance. Overall, kinematic measures revealed distances of the final gap between the mother’s hand and the infant’s head were significantly longer in duration in Scottish pairs than in Japanese pairs at 6 months (**Figure 4A**), which also supports this notion of a more distant, or ‘distal’ care-giving by the former. In contrast, the Japanese mothers brought their torso closer to the infants before final contact with their infant. This gave a longer duration, shorter distance approach with slower speed that altogether produced a gentler, more intimate closure that supports the notion of a more ‘proximal’ care-giving style by Japanese mothers.

The situation was also a playful situation in which the approaching mothers and waiting infants interacted with arm/hand opening for the mothers and leg flailing for the infants together with vocalizations. These behaviors appeared to be “greetings” with expectant arousal and interest given to each other just before the moment of reunion, as the two were beginning to come together. The Scottish pairs appeared to be more strongly motivated to interact with each other.

These greeting behaviors occurred in a zone at around 1 m distance between mother’s hand and infant’s head in both countries. The similarity in the distance among the participants at 9 months in spite of difference in the floor spaces of the experiments in Japan and Scotland (see Materials and Methods) strongly suggests the existence of a common psychological border between two different spaces at about ½ m; the infants were placed on the floor with their legs pointing to their mother, suggesting the 1 m head-hand distance was equivalent to a 0.5 m inter-body distance. Mothers and infants greeted each other when the mother crossed this border on approach. This finding is in agreement with the “peri-personal space” where a multisensory interaction and perceived illusion of tactile and visual sensations of hand occurs immediately around the body (Maravita et al., 2003; Lloyd, 2007). It may be that in the peri-personal space the mother can achieve intimacy and security with her infant even apart from her. The explicit greeting behaviors also might have functioned to help mutual adjustment of the timing of behaviors for the effective pick-up.

For the Scottish it was a playful game-like situation of mutually reading one’s partner’s intentions in their action, and adjusting or attuning one’s behavior during the welcoming return phase as the mother approached to pick-up her infant. This is an everyday-life experience of putting an awake infant to bed and retrieving the infant after sleep, while Japanese infants are almost never forced in such a way to be separated when awake and compelled into sleep (Negayama, 1997). Scottish children in a day nursery were put to sleep while crying with much less bodily contact than in Japan (Negayama and Kawahara, 2010). Thus, Scottish mothers and infants would be accustomed to greet at the reunion, and the infants perhaps developed a habit to complain more noisily at a forced separation.

Evidence indicated Japanese mothers were more empathetic with their infants than their Scottish counterparts during feeding (Negayama, 1998–1999). Having more accommodating and less individualistic traits (Rothbaum et al., 2000), the more contact-seeking of the Japanese pairs might have been inclined to make effort in a more proximal sharing, rather than a distal exchange, of positive emotion. At the same time, it is quite normal for Japanese infants to be laid on a tatami-floor and picked up frequently in the non-sleep context during the course of everyday-life, which is similar to the procedure of the present study. Thus, the separation-reunion by put-down and pick-up may not have been particularly arousing for Japanese infants, and no special motivation for an energetic, positive mutual engagement was encouraged.

This paper expands on how differences in co-regulation and shared expressions of affect, made within culturally specific, embodied and enacted patterns of daily engagement, such as those identified here, establish the early foundations of a cultural anticipation and regulation of affective expressivity within an individual, to be propagated and adapted throughout later childhood and adult life. Learning the expectations and patterns of co-regulation of feelings, and their expressive form manifest in play and everyday rituals of companionship, define and build the character of a community, and its cultural forms of expression (Bruner, 1990; Frank and Trevarthen, 2012).

## CONCLUSION

In this paper, we have identified and measured early features of the regulation of affect, expression, and motor pattern in an everyday embodied intersubjective engagement. We have given evidence to the participatory nature of the interaction from both sides, mother and infant. Future study will help to map the more detailed ontogenesis of a culture, to discern differences in the elements of social expectation, affectivity, and expressivity, and the contribution of both parents and infants to its specific form. Such study may help to elucidate not only the genesis of the cultural form of nations, but also differences in patterning during distress or in cases of pathology. Elucidation of cultural patterns of development is an important route for understanding cognitive as well as socio-emotional development.

## Conflict of Interest Statement

The authors declare that the research was conducted in the absence of any commercial or financial relationships that could be construed as a potential conflict of interest.
